# Effect of Size on the Formation of Solid Solutions in Ag–Cu Nanoparticles

**DOI:** 10.1021/acs.jpcc.2c07132

**Published:** 2023-01-30

**Authors:** Sergiy I. Bogatyrenko, Aleksandr P. Kryshtal, Adam Kruk

**Affiliations:** † V.N. Karazin Kharkiv National University, 4 Svobody Sq., Kharkiv61022, Ukraine; ‡ AGH University of Science and Technology, Al. A. Mickiewicza 30, KrakówPL-30 059, Poland

## Abstract

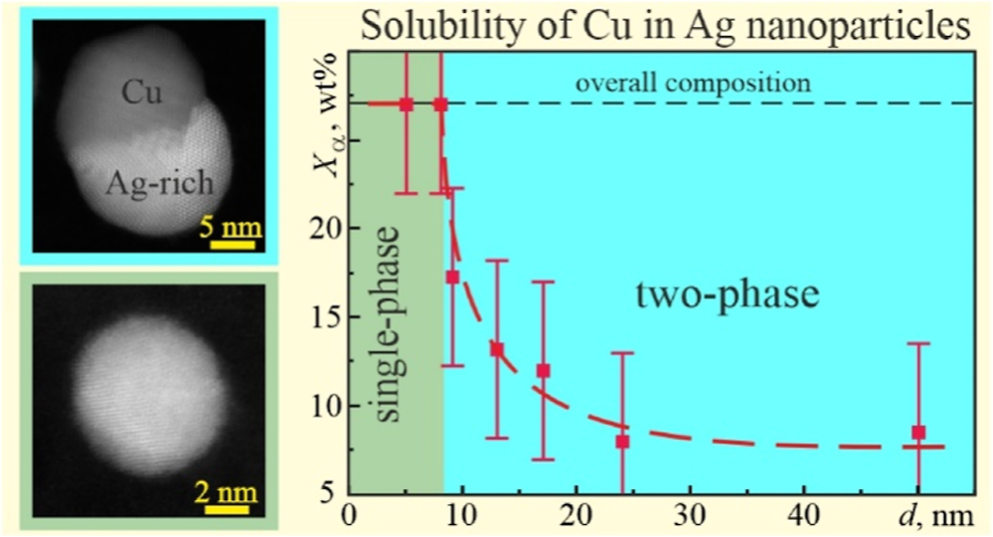

Modern technologies stimulate the quest for multicomponent nanosized materials with improved properties, which are ultimately defined by the atomic arrangement and interphase interactions in the nanomaterial. Here, we present the results of the experimental study of the formation of solid solutions in Ag–Cu nanoparticles in a wide size and temperature range using *in situ* TEM techniques. The Ag–Cu nanoparticles with a eutectic ratio of components were formed on an amorphous carbon film by the physical vapor deposition technique. Electron diffraction, HAADF-STEM imaging, energy-dispersive X-ray spectroscopy, chemical element mapping, and electron energy loss spectral imaging were used for the characterization of mixing patterns and composition of phases in AgCu nanoparticles down to the atomic level. As a result, we constructed the solid-state part of the Ag–Cu phase diagram for nanoparticles with a size down to 5 nm. We found a highly asymmetric behavior of the solvus lines. Thus, the content of Cu in Ag gradually increased with a size reduction and reached the ultimate value for our configuration of 27 wt % Cu at a nanoparticle size below ∼8 nm. At the same time, no Cu-rich solid solution was found in two-phase AgCu nanoparticles, irrespective of the size and temperature. Moreover, a quasi-homogeneous solid solution was revealed in AgCu nanoparticles with a size smaller than 8 nm already at room temperature. A size dependence of the terminal temperature *T*_term_, which limits the existence of AgCu alloy nanoparticles in a vacuum, was constructed. Evaporation of the AgCu phase with the composition of 86 wt % Ag was observed at temperatures above *T*_term_. We show the crucial role of the mutual solubility of components on the type of atomic mixing pattern in AgCu nanoparticles. A gradual transition from a Janus-like to a homogeneous mixing pattern was observed in Ag–Cu nanoparticles (28 wt % Cu) with a decrease in their size.

## Introduction

Nanoparticles consisting of several chemical elements often exhibit superior physicochemical properties compared to elemental nanoparticles, which empower their application in frontier technologies. For instance, Ag–Cu bimetallic nanoparticles are widely used in catalysis,^1−3^ plasmonics,^4^ nanoelectronics,^5^ chemical,^6^ biosensors,^7^ and medicine.^8,9^ Thus, the Ag–Cu system is promising for creating highly active electrocatalysts for the oxygen reduction reaction in fuel cells.^1,2,10−12^ Furthermore, it was shown that the activation energy of the catalytic reaction for the Ag–Cu nanoparticles depends on the atomic mixing pattern. The activation energy of the core–shell structure was significantly lower than that of pure Ag or a homogeneous AgCu alloy.^10^

At the same time, the properties of nanoparticles became size-dependent when their size was reduced below 100 nm. The composition of phases and their arrangement in bimetallic nanoparticles are varied depending on the size, overall composition, heat treatment, and synthesis technique.^13−19^ Both equilibrium and metastable phases and their configurations are observed, which makes theoretical considerations, computer simulations, and experimental studies of single bimetallic nanoparticles an extremely challenging task.

A new direction in thermodynamics, known as nano-thermodynamics, is widely used to tackle the problem.^20−26^ The nano-thermodynamic approach accounts for the surface energy of nanoparticles in the total energy of the system.^22^ In the case of a binary system, a powerful method was developed for predicting the phase diagrams of bulk alloys by computer calculation of phase diagrams (CALPHAD) using experimentally obtained thermodynamic data. This approach simulates studies of solid–liquid phase transformations in nanosized binary alloys and a significant decrease in the melting temperature of the eutectic mixture in several binary systems was shown with the reduction of size.^27,28,33^ The CALPHAD method was further improved in refs (23)(24), by accounting for the effect of the particle’s shape on the surface energy of nanoparticles. Thus, the size evolution of the phase diagram of the Ag–Cu binary system was simulated using a revised CALPHAD-type thermodynamic model.^25,26^ The calculated diagrams were in excellent agreement with the experimental data on the size lowering of the melting temperature in Ag–Cu alloys.

At the same time, computer simulations of the solid-state region of the Ag–Cu phase diagram at the nanoscale are virtually absent. Most of the available calculations of nanosized Ag–Cu phase diagrams using the CALPAD method^23,25,26^ were focused on the melting temperature of alloys. They showed minor to no effect of size on the solid-state solubility of the components. Enhancement of the mutual solubilities of the terminal phases with size reduction was reported in ref (21). Apart from theoretical studies, there are virtually no systematic experimental data on the solid-state solubility in Ag–Cu nanoparticles.

The phase diagram of two-component Janus nanoparticles was simulated using a 3D object stochastic kinetic modeling framework in ref (29). The model enables accounting for nanoparticle shapes and atomic mixing patterns, which is a distinct advantage compared to a regular CALPHAD approach. It was shown that the equilibrium compositions of the phases in Janus nanoparticles change with both the particle’s size and average composition. This means that the use of 3D phase diagrams is required bimetallic nanoparticles.^29^ Furthermore, the shape of the phase diagram depended on the interface structure of the two-phase nanoparticle.

Therefore, this work aims to systematically study the formation of solid solutions in Ag–Cu nanoparticles with a fixed overall composition in the entire range of temperatures using *in situ* heating electron microscopy techniques.

## Objects and Methods

Ag–Cu films with the eutectic ratio of components (28 wt % Cu) and a total mass thickness ranging from 1 to 70 nm were used to study Ag and Cu mutual solid-state solubility. The Ag–Cu system has a eutectic phase diagram with a terminal solid-state solubility *X*_α_ = 8.8 wt % Cu in Ag (α-phase) and *X*_β_ = 8 wt % Ag in Cu (β-phase) in bulk.^34^

The Ag–Cu films were formed on an amorphous carbon film using the PVD technique. The procedure was as follows. The KCl single crystals, used as a substrate, were heated to a temperature of 200 °C and outgassed for 2 h. Then, the substrate was covered with a 2–3 nm-thick layer of amorphous carbon, followed by the silver film deposition. The elevated temperature of the substrate minimized nonequilibrium defects and internal stress in the as-deposited Ag film. The substrate was cooled down to room temperature followed by copper film deposition. The deposition procedure minimized intermixing of Cu on Ag in as-deposited films due to a condensation-retarded diffusion.^40,41^ The mass thickness of the metals was determined using a quartz crystal microbalance (QCM). The condensation rate of 0.5 nm/s was used. Finally, the Ag–Cu films were separated from the substrate by dissolving the salt in distilled water and placed on a dedicated carrier for the *in situ* TEM heating study.

We used two experimental approaches for the *in situ* TEM heating study of Ag–Cu films. First, the selected area electron diffraction (SAED) technique was used to trace the formation of solid solutions in Ag–Cu films over a wide temperature range. The lattice parameters of Ag and Cu differ significantly from each other. They are 0.4088 and 0.3615 nm for Ag and Cu, respectively.^35^ Therefore, the concentration of the solid solution was determined from the lattice constant measurement according to Vegard’s rule.^35^ It should be noted that the SAED technique provides information averaged over ∼1000 square micrometers of the Ag–Ge film. Therefore, the ring patterns represent an average spacing obtained over hundreds of nanoparticles. A very low electron beam current density (∼5 × 10^–6^ A/mm^2^) resulting in a negligible electron dose, which was applied to the specimen, is among the distinct advantages of the diffraction technique.

The experiments were conducted using a transmission electron microscope Selmi TEM—125K operating at 100 kV and fitted with a homemade heating holder.^36^ The film was heated in the temperature range from 20 to 850 °C with steps of 25°. The temperature of the specimen was kept for ∼5 min before acquiring the SAED pattern to ensure the thermodynamic equilibrium of the system.

The positions of the diffraction rings were determined using a free DiffTools plug-in^37^ for Gatan Digital Micrograph software. The well-separated (220) diffraction rings were used to calculate the lattice parameter of the alloys. Then, the data were corrected for pure Ag and Cu thermal expansion coefficients for each system size according to ref (38). The camera length of the microscope was calibrated for each experiment using a reference Al film or MgO single crystals, which ensured the absolute accuracy of the measurements of ±0.002 nm. At the same time, the relative accuracy of the measurements reached the value of ±0.001 nm.

Second, we used high-angle annular dark field detector (HAADF) STEM imaging, energy-dispersive X-ray spectroscopy (EDX) chemical element mapping, and electron energy loss (EEL) spectral imaging (SI) for the characterization of mixing patterns and composition of phases in single AgCu nanoparticles at different temperatures and types of thermal treatment. A probe-corrected FEI Titan G2 60-300 TEM system operating at 300 kV was used. The microscope was equipped with a Wildfire D6 double-tilt heating holder (DENSsolutions), Gatan Image Filter (model 966), and ChemiSTEM EDX system. As a rule, the temperature of the sample was increased in 50 °C steps between 25 and 750 °C. It should be noted that temperatures above 600 °C were applied to the specimen, which was retracted from the pole piece of the objective lens to avoid possible contamination of the TEM system due to the evaporation of the materials. The samples were quenched to 300 °C for TEM characterization in this case.

EDX chemical element maps were acquired over 1000 s at a beam current of 130 pA with a spatial resolution of 0.2 nm per pixel. The composition of the phases was determined from the EDX spectrum averaged over at least 64 pixels to provide a better signal-to-noise ratio. A Cliff–Lorimer standard-less method was used to quantify EDX spectra using K-lines of Ag and Cu. Low-loss EEL spectrum images 66 × 62 pixels in size were acquired with a pixel size of 1 nm and a dwell time of 10 ms. The EELS dispersion of 0.1 eV per channel, convergence, and collection semiangles of 11 and 20 mrad, respectively, were used.

## Results

### Formation of Solid Solutions in Nanosized Ag–Cu Films

When we study the effect of size on a property of a nanomaterial, the bulk system is analyzed first and used as a reference, followed by studies of materials with a smaller size. Here, we used a Ag–Cu film with a total mass thickness of 70 nm as a model of a bulk Ag–Cu system.

**Figure 1:**
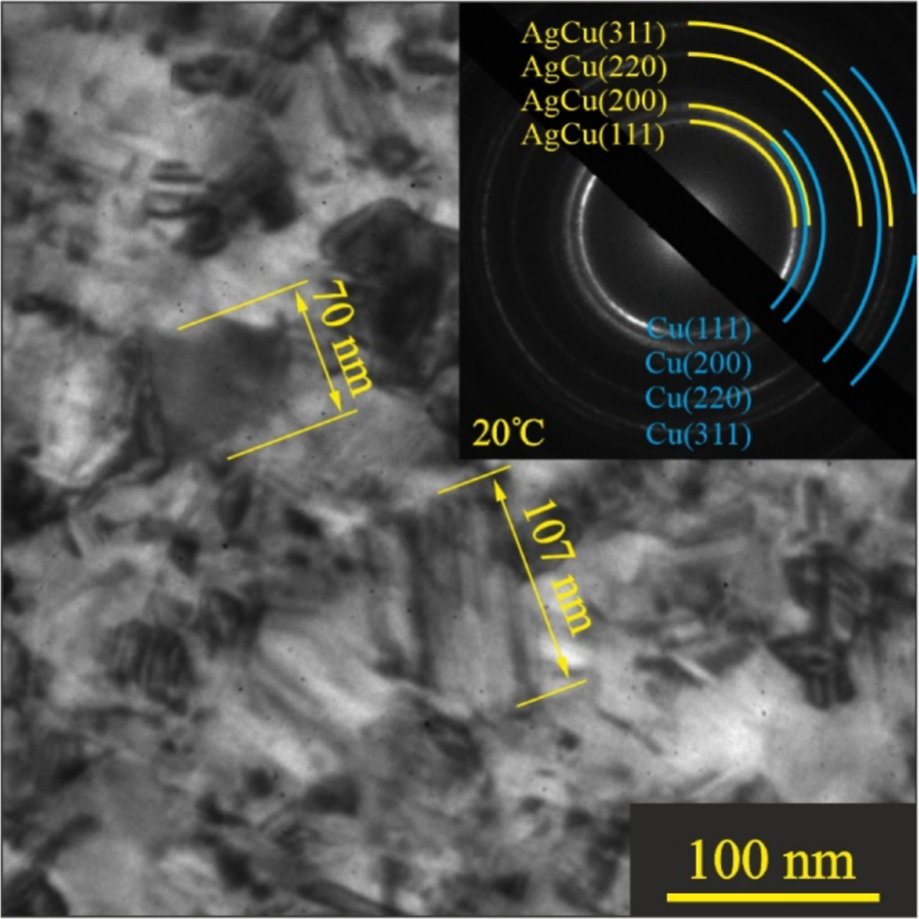
Bright-field TEM image and diffraction pattern (in the inset) of the as-deposited Ag–Cu film with a total mass thickness of 70 nm. The size of the crystallites is shown with a yellow bar.

Figure 1 shows a bright-field (BF) TEM image and SAED pattern (in the inset) of the Ag–Cu film at room temperature. As can be seen, the film was continuous, with a crystallite size of approximately 70–100 nm. The diffraction rings of polycrystalline Cu and Ag were well separated in the SAED pattern, pointing to a bilayer structure of the as-deposited Ag–Cu film.

The measured (220) interplanar distances were 0.144 nm for Ag and 0.129 nm for Cu, which deviates a bit from the reference values of pure Ag (0.1445 nm) and Cu (0.127 nm).^39^ This discrepancy in the lattice constants is likely associated with the formation of nonequilibrium solid solutions at the Ag–Cu interface due to condensation-stimulated effects^41^ already in the as-deposited films. Indeed, the surface of the growing Cu film during the deposition process is a source of excess vacancies. The generated vacancy flow, directed toward the Ag film, changes the ratio of the partial diffusion coefficients of the Ag and Cu. As a result, the Ag partial diffusion coefficient increases while the Cu one decreases. This process induces diffusion of Ag in Cu, resulting in the formation of nonequilibrium solutions at the Ag–Cu interface.

**Figure 2:**
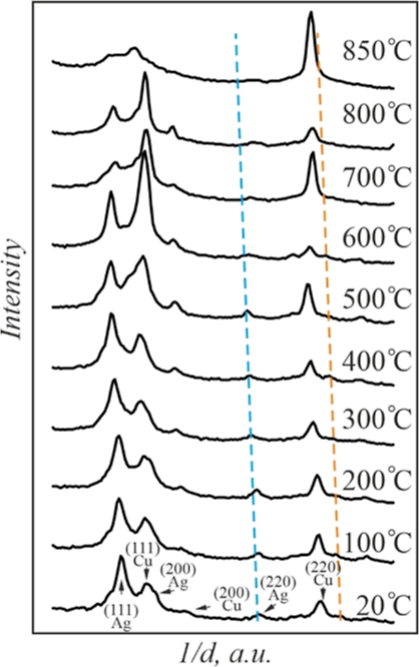
Radially averaged profiles of the SAED patterns of the Ag–Cu film system with a total mass thickness of 70 nm at different temperatures. Dashed lines correspond to the (220) interplanar distances of bulk Ag (blue) and Cu (orange) with the account of the temperature expansion coefficient.

When heated, the positions of the diffraction rings of Cu- and Ag-rich alloys shifted, indicating the variation of compositions of Ag- and Cu-rich solid solutions. Diffraction patterns for this sample at different temperatures are available in the Supporting Information section (Figure S1), while Figure 2 shows the profiles of the SAED patterns for convenience.

An increase of the temperature to 400 °C induced shrinkage of the radii of Cu diffraction rings. Thus, the shift of the (220) diffraction maximum of Cu toward the bulk value is visible in Figure 2. This is due to the decay of the nonequilibrium Cu-rich solid solution, which was formed in the course of the deposition process. Note that the diffraction maxima of Ag remained at the same position.

The positions of the Ag diffraction peaks began to change at a temperature above 400 °C. The Ag(220) diffraction maximum gradually shifted toward the Cu(220) peak with a temperature increase. In contrast, the position of the Cu diffraction maximum was virtually unchanged. This observation points to a gradual dissolution of copper in silver as the temperature rises from 400 to 700 °C. At the same time, the dissolution of Ag in Cu was not registered. The appearance of diffraction spots in the diffraction pattern of the polycrystalline AgCu film was observed at a temperature higher than 750 °C (see Figure S1d), which was associated with the dewetting of the continuous film and the formation of large islands. The diffraction rings of the Ag-rich phase disappeared completely in the SAED pattern at 800 °C, resulting in the diffraction pattern of pure Cu. This temperature is close to the melting temperature of the eutectic mixture in the Ag–Cu system. However, we were not able to register the liquid phase in our specimens.

Furthermore, we did not find Ag in the film anymore; that is, the overall composition of the binary system has changed. Therefore, we referred to this temperature as terminal temperature *T*_term_, and the Ag–Cu system does not exist in a high vacuum at temperatures above *T*_term_. The nature of this temperature will be discussed in detail below.

The lattice parameters of α and β solid solutions were determined from microdiffraction patterns and corrected for the thermal expansion coefficient of bulk metals. Then, the concentration of the solid solutions was calculated according to Vegard’s rule at each temperature studied. The obtained data are presented in Figure 7 in blue color and referred to as bulk. The terminal solubility values of α and β phases were about 8.5 and 5.5 wt %, respectively. Note that the measured data reasonably fit to bulk solvus lines of the Ag–Cu phase diagram, proving the efficiency of the *in situ* diffraction technique for studying mutual solubility in the Ag–Cu system.

We performed similar measurements for Ag–Cu films with a total mass thickness of 10, 7, 4, 2.7, 2, and 1.3 nm to reveal the effect of size on the mutual solubility of Ag and Cu. These films had a distinct island morphology; therefore, the nanoparticles’ size was used instead of the film mass thickness.

The pattern of the solvus lines in Ag–Cu films was similar for thickness values of 10, 7, and 4 nm. Therefore, here we present the data for 4 nm-thick films, while the data for 10 and 7 nm-thick films are available in the Supporting Information section.

**Figure 3:**
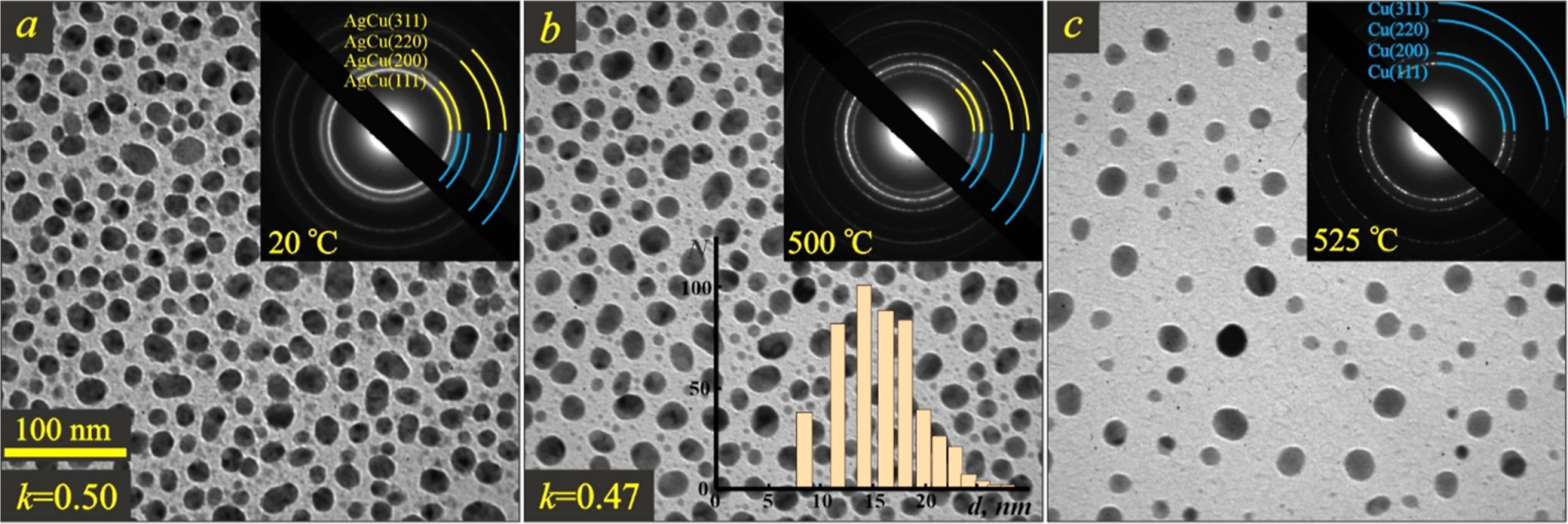
BF-TEM images of the Ag–Cu film with a total mass thickness of 4 nm at 20 °C (a), 500 °C (b), and 525 °C (c). The insets show the corresponding SAED patterns and the size distribution of nanoparticles. The coverage value *k* is indicated in the lower left corner.

Figure 3 shows the morphological (BF-TEM image) and crystalline (SAED patterns are shown in the insets) structure of the 4 nm-thick AgCu film at different temperatures. The as-deposited film had an island structure with a coverage *k* (*i.e.*, the ratio of the projection area of the particles to the total substrate area) of approximately 0.5 (Figure 3a).

Note that the particle’s shape deviated from circular symmetry. Therefore, we used an equivalent particle size to characterize the AgCu system’s size. The equivalent size was defined as the diameter of a spherical particle having the same surface-to-volume ratio as that of the irregular-shaped island of the film. When the mass thickness of the film *t* (as measured by QCM) and the coverage value *k* are known, the mean height of the islands *h* could be assessed using the equation *h* = *t*/*k*, which gave *h* ≈ 8 nm. The footprint diameter of the nanoparticles varied in the range of 12–18 nm, as follows from the distribution chart (inset in Figure 3b). Hence, the islands had a shape of a truncated ellipsoid, and the equivalent diameter of these nanoparticles was equal to ∼13 nm. The nanoparticle’s size and shape remained virtually unchanged up to a temperature of 500 °C (Figure 3b), and only a minor decrease in the coverage was registered. The evaporation process was activated at a terminal temperature of 525 °C for 13 nm-size AgCu nanoparticles.

**Figure 4:**
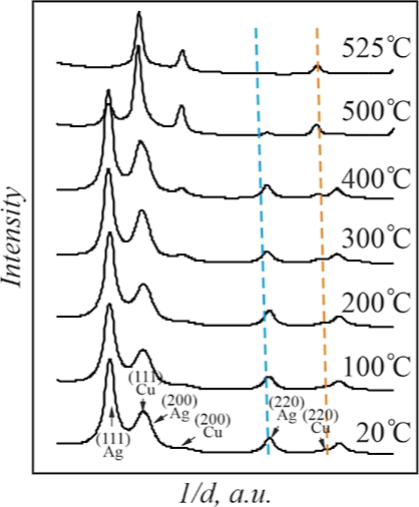
Radially averaged profiles of the SAED patterns of the Ag–Cu film system with a total mass thickness of 4 nm at different temperatures. Dashed lines correspond to (220) interplanar distances of the Ag (blue) and Cu (orange) nanoparticles with the size of 13 nm.^38^

Figure 4 shows the profiles of the SAED patterns of this specimen in the 20–525 °C temperature range. Note a progressive shift of the (220) ring position of the Ag-rich phase in nanoparticles with temperature increase concerning that of pure Ag (blue) nanoparticles of the same size.^38^ This observation proves a gradual dissolution of Cu in Ag with a temperature increase. The terminal solubility of Cu in 12 nm Ag islands reached the value of 13 wt % at *T* = 500 °C.

The rings of Ag vanished when the temperature reached 525 °C, as seen in Figure 4. This observation is in line with the TEM data shown in Figure 4.

At the same time, we did not register Cu-rich solution in the nanoparticles. The copper diffraction maxima were almost unresolved in the diffraction patterns up to a temperature of 300 °C. A negligible concentration of Ag in Cu was registered in the 300–500 °C temperature range. At a temperature of 500 °C, the positions of Cu rings corresponded to that of pure Cu.

The obtained data are presented in Figure 7, along with the results of the measurements for 10 and 7 nm-thick Ag–Cu films. One can see that the equilibrium solubility of Cu in Ag progressively increases with a reduction of the nanoparticles’ size down to 13 nm (Figure 7).

**Figure 5:**
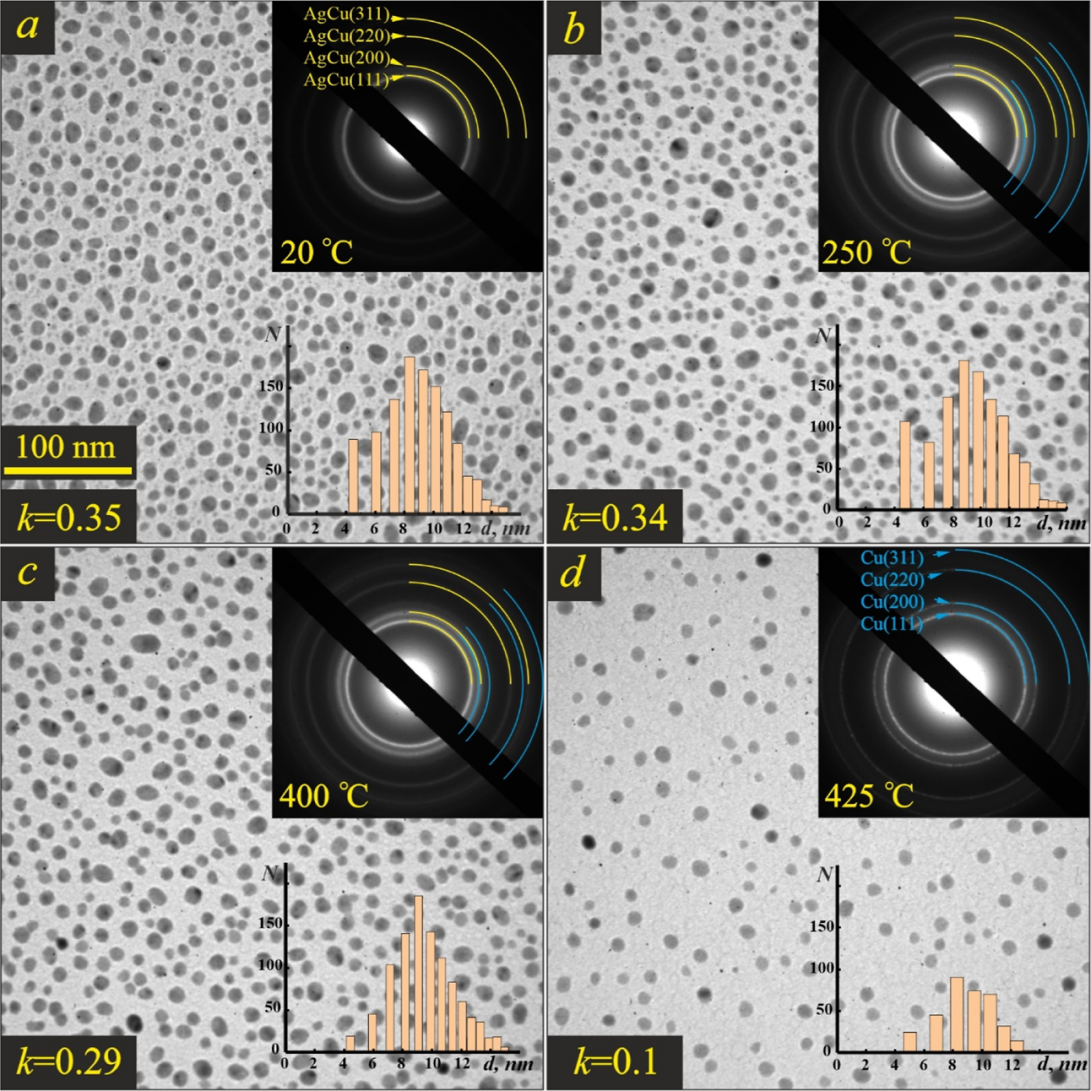
BF-TEM images of the Ag–Cu film with a total mass thickness of 2 nm at 20 °C (a), 250 °C (b), 400 °C (c), and 425 °C (d). The insets show the corresponding SAED patterns and the size distribution of nanoparticles. The coverage value is indicated in the lower left corner.

**Figure 6:**
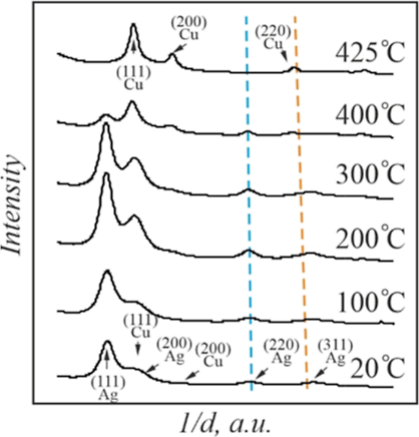
Radially averaged profiles of the SAED patterns of the Ag–Cu film system with a total mass thickness of 2 nm at different temperatures. Dashed lines correspond to (220) interplanar distances of the Ag (blue) and Cu (orange) nanoparticles with a size of 7.5 nm.^38^

The formation of solid solutions in thinner films (with a total thickness of 2.7, 2, and 1.3 nm) differs substantially. Thus, Ag-rich solid solution (α-phase) with a composition close to the as-deposited one was observed already at room temperature. Naturally, we did not find the Cu-rich phase in the nanoparticles. However, a pure Cu was registered at the terminal temperature. The results of the measurements for the Ag–Cu film with a mass thickness of 2 nm are presented in Figures 5 and 6. The data for 1.3 and 2.7 nm-thick films are available in the Supporting Information section.

The as-deposited AgCu film with a mass thickness of 2 nm had an island structure with a coverage *k* of approximately 0.35 (Figure 5a). Hence, the mean height of the nanoparticles was about 6 nm. The footprint distribution of the nanoparticles had a wide maximum at 6–11 nm (inset in Figure 5a). Therefore, the equivalent diameter of the nanoparticles in this film was assessed to ∼8 nm.

The diffraction pattern of the as-deposited Ag–Cu film showed the rings of a fcc Ag-rich alloy while the Cu ones were absent (inset, Figure 5a). The lattice parameter of the α phase corresponded to a 23 wt % Cu alloy, which was somewhat smaller than that of the as-deposited composition of the film. A minor change in the lattice parameter was registered in the 20–400 °C temperature range as it is proven by the profiles of the SAED patterns of the Ag–Cu film (Figure 6). The composition of the α phase grew up to 27 wt % Cu and the complete mixing of Ag and Cu occurred at 400 °C, though the change in the composition was within the error of the measurements.

**Figure 7:**
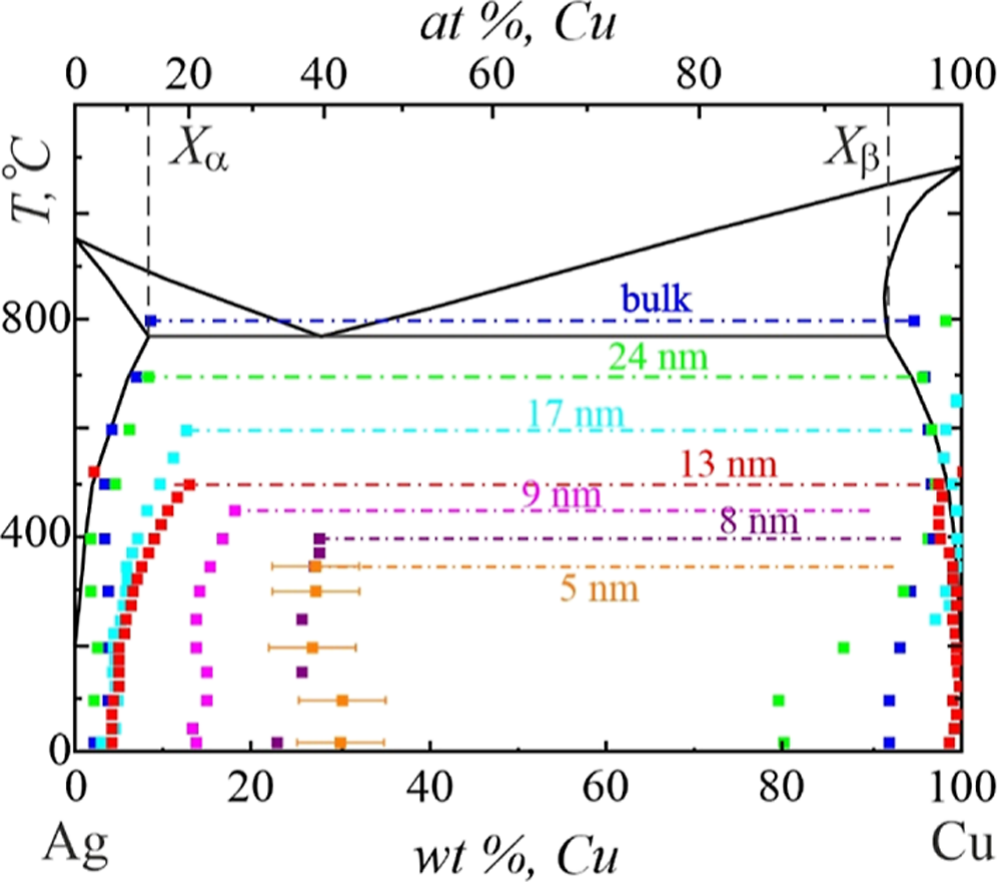
Phase diagram of Ag–Cu nanoparticles with different sizes.

The shape and size of AgCu islands remained practically unchanged up to a temperature of 250 °C, as follows from the TEM image and size distribution chart (Figure 5b). Some coarsening of a few nanometer-size Cu islands (barely visible in Figure 5a), which were located in the open areas of the carbon film, was observed (Figure 5b). This is due to the activation of surface diffusion^44^ and Oswald ripening.^45^ These processes result in the appearance of a weak and diffuse Cu diffraction ring in the SAED pattern at 250 °C along with the rings of the Ag-rich alloy (inset, Figure 5b). The results of the measurements of α phase composition as a function of temperature are shown in Figure 7.

Noticeable changes in both morphological and crystalline structures of the film occurred at a temperature above 400 °C (Figure 5c). The (200) copper diffraction ring (inset, Figure 5c) became intense and narrow, and its position corresponded to that of pure Cu. At the same time, the number of islands with a diameter below 6 nm drastically decreased at 400 °C. In contrast, the number of 10 nm-size islands remained virtually unchanged, pointing to the activation of the evaporation process. This process intensified at 425 °C, resulting in a substantial reduction in coverage (Figure 5d) and the complete disappearance of the Ag-rich alloy rings in the diffraction pattern (inset in Figure 5d). In the end, rare nanoparticles of pure Cu remained in the film.

Similar behavior was observed for AgCu films with a mass thickness of 1.3 nm (Figure S2). In this case, the equivalent size of the nanoparticles, assessed from the distribution chart and the coverage, amounted to ∼5 nm. As in the previous case, the Ag-rich solid solution was already formed in the as-deposited film and remained virtually unchanged until a terminal temperature of 350 °C was reached. At this temperature, the smallest nanoparticles disappeared, fading the diffraction rings of the Ag-rich alloy and leaving pure Cu nanoparticles in the film (Figure S2b). It should be noted that slightly overestimated values of the alloy concentration in the as-deposited films likely result from the nonequilibrium nature of the deposition process, as already discussed.

Figure 7 summarizes the obtained data on the mutual solid-state solubility of Ag and Cu in nanoparticles. As a main result, the solvus lines of the phase diagram of Ag–Cu nanoparticles with an overall composition of 28 wt % Cu were constructed for sizes down to 5 nm. Note the asymmetric shift of the solvus lines of the phase diagram. Thus, the solubility of Cu in Ag significantly increases with the reduction of the size of the binary system. At the same time, Ag was practically insoluble in Cu, irrespective of the size and temperature.

■ equilibrium Ag–Cu phase diagram,^34^ ■ (blue)—experimental data for bulk, ■ (green)—24 nm, ■ (red)—12.5 nm, ■ (pink)—9 nm, ■ (purple)—7.5 nm, and ■ (orange)—5.5 nm-size nanoparticles. The terminal temperature is shown with dotted–dashed lines. The experimental measurement errors which are shown for the size of 5 nm correspond to all measurements.

However, the BF-TEM images and electron diffraction patterns provide limited information on the composition of single nanoparticles. The following questions should be addressed for further insight into phase transformations in Ag–Cu alloy nanoparticles.

1. How are Ag and Cu distributed within a single nanoparticle?
2. What is the nature of a terminal temperature *T*_term_, and what is the composition of the evaporated phase?

We used analytical STEM to study solid solutions in single AgCu nanoparticles to tackle these problems.

### Atomic Mixing Pattern of AgCu Nanoparticles

**Figure 8:**
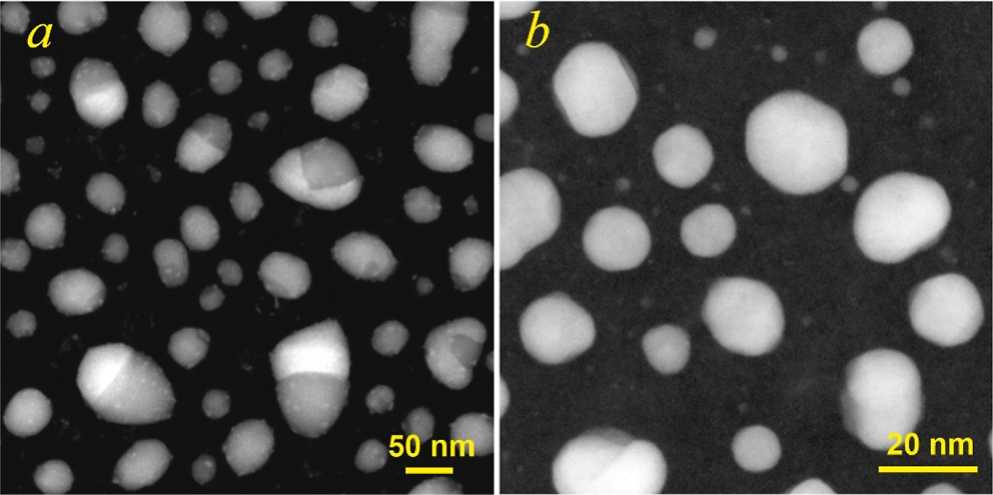
HAADF-STEM images of the Ag–Cu film with a mass thickness of 7 nm (a) and 2.7 nm (b) after a thermal treatment.

**Figure 9:**
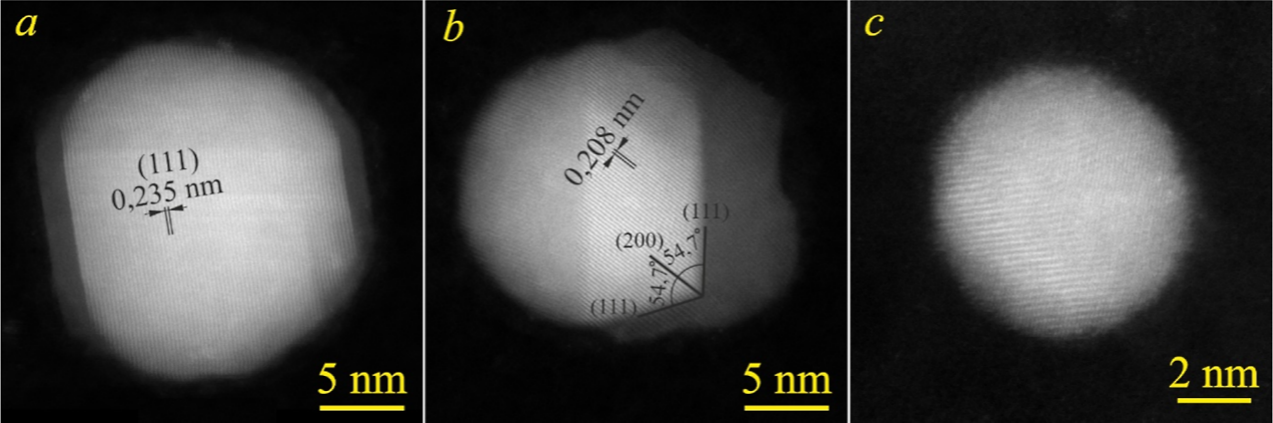
High-resolution HAADF-STEM images of phase-separated (a,b) and single-phase (c) Ag–Cu nanoparticles.

Figure 8 shows the HAADF-STEM images of Ag–Cu films of different thicknesses (with a different mean size of nanoparticles) after the diffraction studies. Janus-like and single-phase nanoparticles were firmly detected in the *Z*-contrast images due to a difference in the atomic number of Ag (*Z* = 47) and Cu (*Z* = 29). Janus morphology, consisting of Ag- and Cu-rich phases, dominated in nanoparticles with a size larger than ∼8 nm. The dihedral angles at a three-phase junction in Janus nanoparticles varied widely due to different volume fractions of the phases, size, and combinations of surface and interfacial energies. As a result, “ideal” Janus nanoparticles with two halves separated by a flat interface along a diameter, “side-segregated”, and “crescent-like” Janus structures were observed (Figures 8a and 9a,b). Careful analysis of high-resolution images revealed that the α/β interface was preferentially coincident with the (111) planes of silver (Figure 9a,b), which are likely to provide the lowest energy path for copper dissolution and segregation.

On the other hand, the nanoparticles with a size below ∼8 nm were mostly a single-phase AgCu alloy (Figure 8b). Figure 9c exemplifies the high-resolution HAADF-STEM image of alloy nanoparticles with an almost uniform mixture of Cu and Ag atoms. The EDX spectrum from this nanoparticle is shown in the Supporting Information section (Figure S3). Its quantification gave about 30 wt % of Cu, which was close to the as-deposited ratio of metals. Hence, Cu and Ag were completely dissolved in nanoparticles with a size below ∼8 nm, forming a quasi-homogeneous solid solution.

In summary, a gradual transition from Janus-like to a mixed pattern was observed in Ag–Cu nanoparticles (28 wt % Cu) with a decrease in their size.

**Figure 10:**
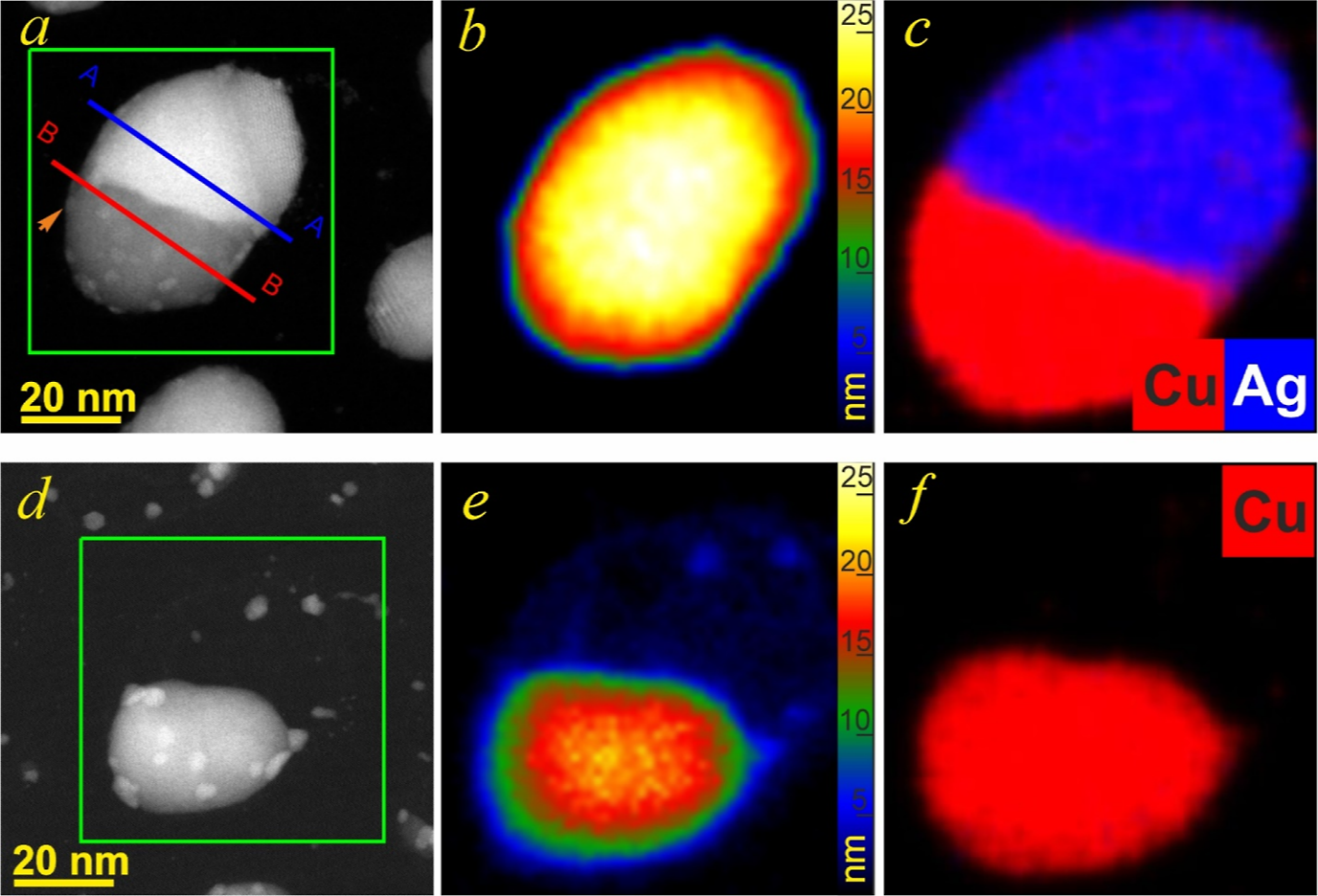
HAADF-STEM images of Ag–Cu nanoparticles annealed to 600 °C (a) and 750 °C (d) along with false-color thickness maps (b,e) and EDX composite maps (c,f) of Cu (red) and Ag (blue) acquired from the regions marked by green rectangles. AA and BB lines show the position of cross-sections, which were used for assessing the parameters of the phases. Arrow indicates the CuO nanoparticle.

We used EDX chemical element mapping for the quantification of the composition of solid solutions in single nanoparticles. Figure 10 shows a Janus nanoparticle with a size of ∼30 nm, along with the EDX chemical element maps. EDX analysis confirmed that Janus nanoparticles consisted of almost pure Cu and Ag-rich phases. The mean composition of the α phase was ∼7.5 wt % of Cu, which is slightly lower than a terminal solubility *X*_α_ for such a size of nanoparticles. The mean content of Ag in the β phase did not exceed 1 wt %. Note that Ag and Cu are almost insoluble at room temperature, according to the phase diagram of bulk alloys.^34^ Therefore, EDX data confirmed the Janus morphology of nanoparticles.

At the same time, they showed that complete phase unmixing of the Ag-rich alloy did not happen under cooling. Furthermore, no Ag surface segregation was noticed in the Cu-rich part of the nanoparticles, though it is often reported in the literature^13−16^ due to a smaller surface energy of Ag than Cu.^17−19,67^ This is additional proof of the insolubility of Ag in Cu in Janus nanoparticles with an overall composition close to the eutectic one.

### Terminal Temperature

To unravel the nature of terminal temperature *T*_term_, we assessed the composition of the evaporated phase by analyzing the overall composition and volume of a single nanoparticle before and after annealing to evaporation temperature. We used an *in situ* heating TEM approach to characterize the very same nanoparticle.

Figure 10 shows the Janus nanoparticle, which was used in the analysis. The nanoparticle was annealed to 600 °C to reach the equilibrium shape (Figure 10a), followed by annealing to an evaporation temperature of 750 °C (Figure 10d). To reconstruct the volume of the nanoparticle, one needs to know its 3D shape, which is challenging from 2D TEM images. Therefore, the EEL SI technique was used to assess the height of the nanoparticle and the size of the wetting zone. In this technique, the focused electron beam scans over a selected region, and the low-loss EEL spectrum is acquired at each raster point. The relative thickness *t*/λ (where *t* was the thickness, and λ was the mean free path of the electron) was assessed by using a log-ratio method from the ratio of the zero-loss electrons to the total transmitted intensity.^46^ After the removal of the substrate component, the thickness map of the AgCu nanoparticle was assessed (Figure 10b). We used the mean free path of the electron λ = 137 nm for Cu and λ = 132 nm for AgCu alloy, according to Jakubovski.^47^

Table 1 accumulates the measured parameters of the nanoparticle under study. The width and length of Ag and Cu domains were measured from images, the composition was assessed from EDX spectra, and the height was determined from EELS data.

**Table 1:** Parameters of the Ag–Cu Nanoparticle (Figure 10a,d) Were Determined from the HAADF-STEM Images, ELLS, and EDX Data

	before		after
parameter	Ag side	Cu side	Cu
content of Cu, wt %	7.5	100	100
width *d*, nm	37.6	36	26
length *l*, nm	29.4	24.6	36
height *H*, nm	27	27	23
diameter of the contact area, *z*, nm	26	21	23
Θ, degree	147	132	148
volume *V*, nm^3^	16 812	13 914	12 266

**Figure 11:**
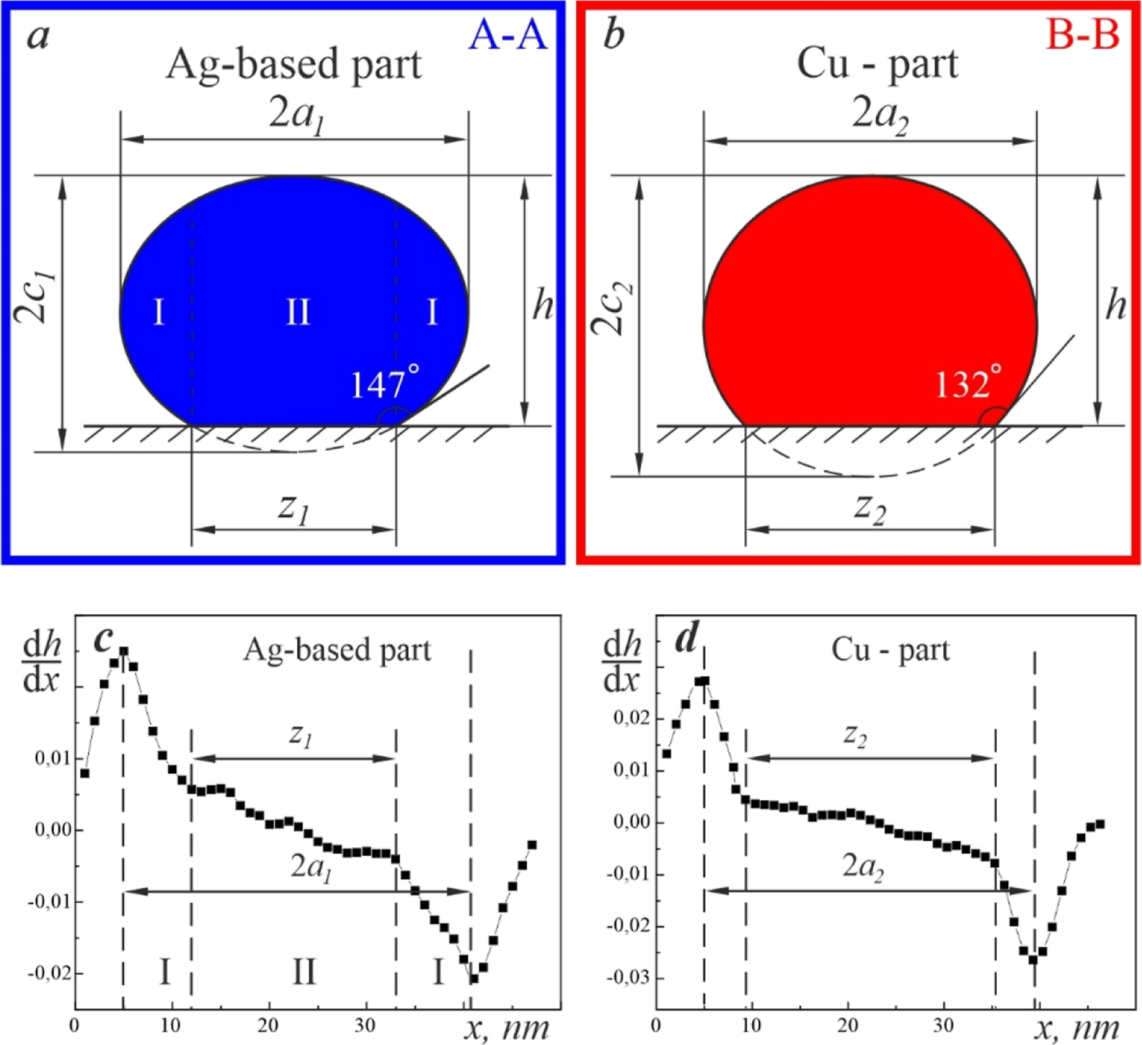
A schematic view of cross-sections A–A (a) and B–B (b) of Ag and Cu domains of the Janus nanoparticle is shown in Figure 10a. (c,d) are the derivatives of EELS SI data (Figure 10b) along the corresponding cross-sections.

Since the height of the particle was higher than half of the width, then in the first approximation, we assumed that the AgCu nanoparticle had a shape of a truncated ellipsoid with the interphase boundary lying along the plane normal to the substrate. The volume of the truncated ellipsoid was assessed using the expression , where *a*, *b*, and *c* are the semiaxes of the ellipsoid, and *h* is the height of its cap. In our case, *a* = *d*/2, *b* = *l*, and *h* = *H*. However, to correctly reconstruct the shape of the ellipsoid, one needs to know the semiaxis *c*, which could be determined from the contact angle or the size of the wetting zone (Figure 11a).

We were inspired by the photometric approach, which was used in the past for measuring the wetting angle of nanoparticles from TEM photographic plates.^48^ The thickness of a nanoparticle has two distinct regions along the diameter. Thus, in area I of the nanoparticle (in Figure 11a), the thickness shows significant variation along the *x*-axis. In contrast, the thickness in region II is almost constant. Therefore, the slope of the first derivative of the height must have an inflection point at the triple point. Figure 11c,d shows the d*h*/d*x* plots along the A–A (a) and B–B (b) cross-sections shown in Figure 10.

The inflection points of the curve allow one to measure the diameter of the wetting zone *z*. As a result, the semiaxis *c* and the contact angle Θ were determined for the Ag and Cu domains of the nanoparticle under study (Table 1). With these data, the volume of Ag and Cu domains was calculated, and the mass of the AgCu nanoparticle *m*_AgCu_ = 2.98648 × 10^–16^ g was obtained. We used density values 10.455 and 8.92 g/cm^3^ for the Ag-7.5 wt % Cu alloy and Cu, respectively.

The shape and volume of the nanoparticle, which was annealed to 750 °C (Figure 10d), were determined in the same way, and the results are presented in Table 1. The particle contained only pure copper, and its mass *m*_Cu_ was equal to 1.09412 × 10^–17^ g.

With these data, the composition and mass of the evaporated material of the nanoparticle were assessed. The calculations showed that the evaporated phase consists of both Ag and Cu with a mean composition of about 86 wt % Ag and 14 wt % Cu.

It should be noted that a few nanometer-size particles, which are visible in the images (*e.g.*Figure 10d), resulted from partial oxidation of Cu in the residual atmosphere of a TEM system at a high temperature. Thus, Figure S4 in the Supporting Information shows the Fourier spectrum of the high-resolution image of the nanoparticle marked by the orange arrow in Figure 10a, which reveals a crystalline structure of fcc CuO. CuO nanoparticles have a melting temperature of about 1227 °C^39^ and are inert to Ag and Cu at the temperatures used in the study.

## Discussion

The results of the experimental study enabled the construction of the phase diagram of Ag–Cu nanoparticles with a mean composition of 28 wt % of Cu in a vacuum over a wide range of temperatures and sizes (Figure 7). The AgCu liquid phase did not exist in a high vacuum; therefore, the phase diagram of Ag–Cu nanoparticles was limited to the solid region only. Furthermore, we investigated alloying in bimetallic nanoparticles. Consequently, the constructed phase diagram should be referred to as the solubility diagram of Ag–Cu nanoparticles with a fixed composition. The composition of solid solutions in phase-separating nanoparticles did not coincide with solubility lines and is the subject of ongoing research.

We showed that the formation of solid solutions in Ag–Cu nanoparticles substantially differs from those in bulk alloys. It has a highly asymmetric behavior with size reduction. Thus, a gradual increase of Cu solubility in Ag resulting in a shift of the solvus line in the Ag–Cu phase diagram and shrinkage of the miscibility region is obvious in Figure 7. The terminal concentration *X*_α_ of 13 wt % Cu was reached at 500 °C in the Ag-rich alloy nanoparticle with a size of 13 nm, which is in reasonable agreement with refs (30) and (49).

At the same time, the reverse effect of size was registered for the Cu-rich alloys. Though the 5.5 wt % of Ag was found in the bulk Cu-rich phase at ∼800 °C, the concentration of Ag decreased sharply with size reduction. Thus, the average concentration of Ag dissolved in the Cu domain of 13 nm-size Janus nanoparticles did not exceed 2 wt % at a terminal temperature of 500 °C, which is within the experimental measurement error. Hence, Cu preferably dissolves in Ag in two-phase nanoparticles with the hypoeutectic overall composition.

This observation is in line with the results of Monte Carlo simulations of the phase diagram of Ag–Cu nanoparticles of various shapes, which showed a stronger increase in solubility for Cu in Ag than for Ag in Cu.^64^ Furthermore, the diffusion coefficient of copper into silver at 700 °C is almost twice that of the silver one into copper (8.3 × 10^–11^ and 4.37 × 10^–11^ cm^2^/s, respectively);^65^ therefore, copper should predominantly diffuse into silver, under equal conditions. On the other hand, our data do not correlate with the results of ref (50), which reports a preferential dissolution of Ag in Cu in Janus nanoparticles. Thus, a concentration of 5.8 wt % Ag in the Cu-rich phase was reported in ∼50 nm-size nanoparticles at 520 °C. The main difference between Ag–Cu nanoparticles, studied in ref (50) and in the present work, was the overall composition of the nanoparticle, namely, 43 wt % Cu and 28 wt % Cu, respectively. Therefore, the overall composition of the Janus nanoparticle governs the dominant type (α or β) of solid solution. This is in line with the results of computer simulations of ref (29), which showed that the equilibrium concentrations of solid solutions depend on the average composition of the Janus nanoparticle.

Another work finding revealed the formation of a quasi-homogeneous solid solution in AgCu nanoparticles smaller than 8 nm at room temperature. This size is commonly referred to as a critical size, and the components of a binary system which are partly soluble or insoluble in the solid state become fully miscible. The concentration of Cu in a Ag-rich solid solution reaches approximately 27 wt % at the size of 8 nm, which was the as-deposited composition of the films. At this size, a transition between Janus and the single-phase morphology of the nanoparticles occurred (Figure 9). In other words, when the components’ solubility reaches the overall composition of the system, the single-phase nanoparticles are formed.

The existence of critical size was reported for other binary systems, for example, Au–Ni, Au–Co, and others.^31,32,51,52^ A critical size of 5 nm was reported for Ag–Cu nanoparticles in the concentration range of 10–70 wt % Ag;^49^ that is, a solid solution was formed below this size during condensation at room temperature of the substrate. A thermodynamic background for the alloying ability of bimetallic nanoparticles was proposed in ref (53). It was shown that both the formation enthalpy and formation Gibbs free energy decrease with the decrease of particle size, which means that Cu and Ag prefer to become an alloy as the size becomes extremely small. This critical size ensuring alloying of Cu and Ag in the entire composition range was assessed to be ∼2 nm.

The experimentally found size of 8 nm was larger than that predicted theoretically. We suppose several reasons for this discrepancy. First, we proved the complete alloying of Ag and Cu in a limited composition range, that is, from 0 to 28 wt % of Cu. In this concentration range, the alloy formation enthalpy curve has a minimum, which favors complete alloying at larger than 2 nm sizes. On the other hand, the alloy, which was formed during the deposition, might be a “kinetically trapped” one so that phase separation does not occur. Thus, no phase separation in the AgCu alloy happened when the size of nanoparticles was below a critical value of 5 nm.^49^ This size was associated with the spinodal wave assessed according to the Cahn–Hilliard theory.

At the same time, if the composition of the alloy lies in the region of the phase diagram bounded by spinodal and bimodal lines, then unmixing of a metastable alloy occurs *via* a nucleation and growth mechanism, which requires the creation of a viable nucleus of a critical size. In this case, the nucleus size might be larger than the size of AgCu nanoparticles, and the nucleation process will not occur. We observed similar critical phenomena upon the melting of Au/Ge, Ag/Ge, and Sn/Bi films, where the films with a metal thickness below a threshold value did not melt at the eutectic temperature of the bulk alloy.^33,54−56^ Furthermore, nucleation phenomena in a finite nanoparticle volume are constrained due to the depletion effect.^57^

The factors mentioned above facilitate retaining a quasi-homogeneous solid solution in AgCu nanoparticles up to a size of 8 nm.

The phase diagram shown in Figure 7 has a terminal temperature specific for each size of the nanoparticles. Intensive evaporation of the nanoparticles was activated at this temperature, ultimately resulting in pure Cu remainders. Three options seem possible, namely, (i) sublimation of Ag from the alloy due to its lower vapor pressure as compared to Cu,^42,43^ (ii) congruent sublimation of AgCu alloy, and (iii) eutectic melting^34^ followed by evaporation of the liquid phase.

Our assessments showed that the composition of the evaporating phase was about 86 wt % Ag–Cu. This composition did not directly fit any of the abovementioned options.

**Figure 12:**
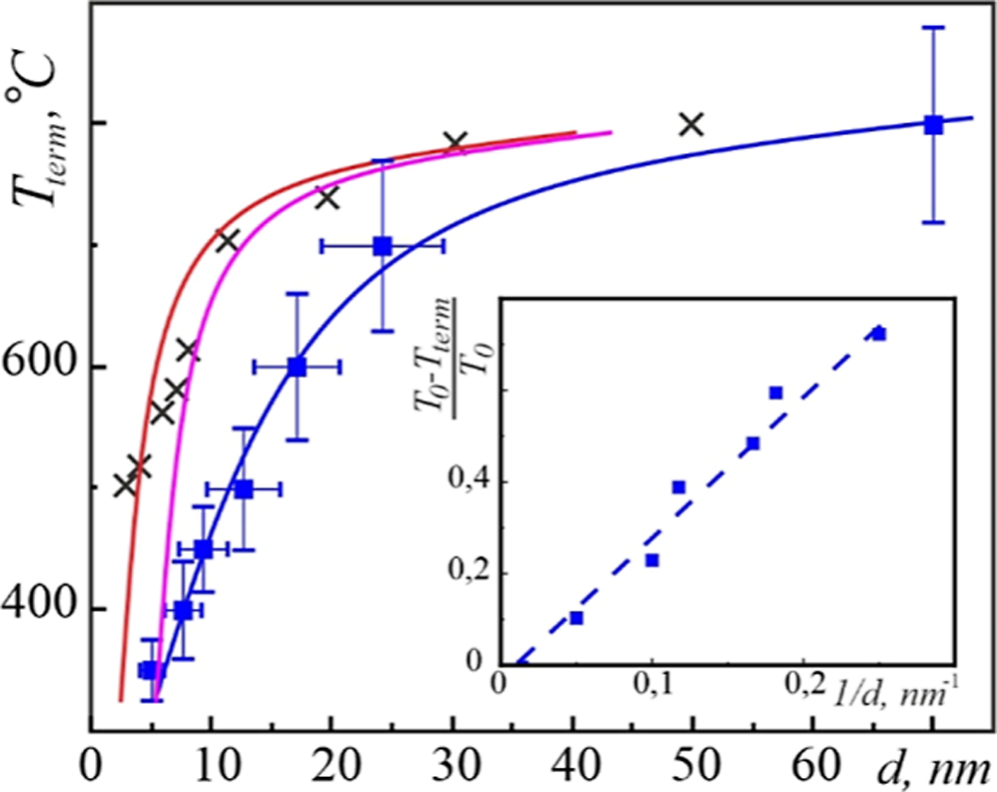
Temperature *T*_term_ [■ (blue)] in AgCu nanoparticles as a function of size along with literature data on the eutectic temperature in the Ag–Cu system (pink line^25^ and red line^26^) and sublimation temperature of Ag nanoparticles (×).^43^ The inset shows the relative *T*_term_ in inverse size coordinates with a linear least-square fitting.

Figure 12 shows *T*_term_ temperature as a function of the AgCu alloy nanoparticle size. Please note that the relative temperature  is directly proportional to the inverse size of the nanoparticles as it is expected for the size-dependent thermodynamic properties of materials.^66^ Here, *T*_0_ is the terminal temperature of the 70 nm-size particles, which were used as a bulk reference. If we compare the measured values of *T*_term_ with the sublimation temperature of pure Ag nanoparticles *T*_subl_(25,26,43) (Figure 12), then for the majority of sizes, *T*_term_ < *T*_subl_. On the other hand, the dissolution of copper atoms in the silver lattice raised the surface energy of the silver.^58^ As a result, the binding energy of the surface atoms of silver decreased, making its sublimation from the AgCu alloy easier than that of pure Ag. At the same time, this fact cannot explain the evaporation of 14 wt % Cu along with Ag.

Therefore, eutectic melting followed by evaporation of the liquid phase should accompany the sublimation of Ag. The eutectic composition in nanoparticles is unknown, and it may differ significantly from that given by the equilibrium phase diagram. Thus, the CALPHAD assessment of the Ag–Cu phase diagram showed a shift of the eutectic composition toward the Ag side with a reduction of particle size and a size lowering of the eutectic temperature.^25,59^ For example, the eutectic composition of ∼10 nm-size AgCu nanoparticles was assessed to be 10 wt % of Cu. Furthermore, there is no guarantee of congruent evaporation of the liquid eutectic phase, that is, the compositions of the liquid and the vapor phases being in equilibrium can differ from each other.^60,61^ At the same time, the eutectic temperature of Ag–Cu nanoparticles, which was assessed using the CALPHAD approach,^25,26^ was somewhat higher than the *T*_term_ temperature (Figure 12). This could be due to the surface premelting effect, which preceded the eutectic melting in binary alloy nanoparticles.^62,63^

Hence, we suppose two evaporation processes occur simultaneously at *T*_term_ temperature, namely, the primary one, which forms a liquid phase at the specific surface planes of the nanoparticle with subsequent evaporation, and the secondary one, which is the sublimation of Ag.

## Conclusions

The formation of solid phases in Ag–Cu nanoparticles with 28 wt % Cu was studied using in situ TEM techniques in wide size and temperature ranges. The outline of the phase diagram of Ag–Cu nanoparticles was experimentally constructed as a function of the particles’ size. We found that the solvus line on the Ag side gradually shifts to a higher content of Cu, reaching the ultimate for our configuration value of 28 wt % Cu at a size below ∼8 nm. At the same time, no Cu-rich solid solution was found in AgCu nanoparticles with a Janus mixing pattern, irrespective of the size and temperature.

The formation of a quasi-homogeneous solid solution of about 27 wt % Cu was revealed in AgCu nanoparticles with a size smaller than 8 nm already at room temperature. The alloy in these AgCu nanoparticles preserved the single-phase structure in the entire temperature range, that is, from room to terminal temperature.

A terminal temperature, which limits the existence of AgCu alloy nanoparticles in vacuum, was established. This temperature is specific for each size of the nanoparticles, gradually decreasing from 800 to 350 °C with size reduction. Intensive evaporation of AgCu nanoparticles was found at temperatures above the terminal one, and the composition of the vapor phase was assessed to 86 wt % Ag.

We showed that the mixing pattern of AgCu nanoparticles with the overall composition of 28 wt % Cu gradually transforms from Janus-like to a single-phase structure with a decrease of the size, which is governed by the mutual solubility of components.

## Supporting Information Available

SAED patterns of the Ag–Cu film with a mass thickness of 70 nm at different temperatures; BF-TEM images of the Ag–Cu film with a total mass thickness of 1.3 nm at 20 and 375 °C along with the corresponding SAED patterns; EDX spectrum from the single-phase Ag–Cu nanoparticle shown in Figure 10c; and HAADF-STEM image of Ag–Cu Janus nanoparticles (PDF)
