# Supplementary material for: Effect of Size on the Formation of Solid Solutions in Ag–Cu Nanoparticles
Source: J Phys Chem C Nanomater Interfaces. 2023 Jan 30;127(5):2569–80. doi: 10.1021/acs.jpcc.2c07132 (PMC9931174; doi:10.1021/acs.jpcc.2c07132)
Supplement: Supplementary file 1 — jp2c07132_si_001.pdf [file jp2c07132_si_001.pdf]

Supporting information

Effect of Size on Formation of Solid Solutions in Ag-Cu Nanoparticles

Sergiy I. Bogatyrenko[a], *Aleksandr P. Kryshtal*[b], *Adam Kruk*[b]

[a] V.N. Karazin Kharkiv National University, 4 Svobody sq., 61022 Kharkiv, Ukraine

[b] AGH University of Science and Technology, Al. A. Mickiewicza 30, PL-30 059 Kraków, Poland

Corresponding Authors

Sergiy I. Bogatyrenko − V.N. Karazin Kharkiv National University, 4 Svobody sq., 61022 Kharkiv, Ukraine; http://orcid.org/0000-0002-6044-6886; Email: sib@univer.kharkov.ua

Aleksandr P. Kryshtal − AGH University of Science and Technology, Al. A. Mickiewicza 30, PL-30 059 Kraków, Poland; http://orcid.org/0000-0002-6528-8821; Email: kryshtal@agh.edu.pl

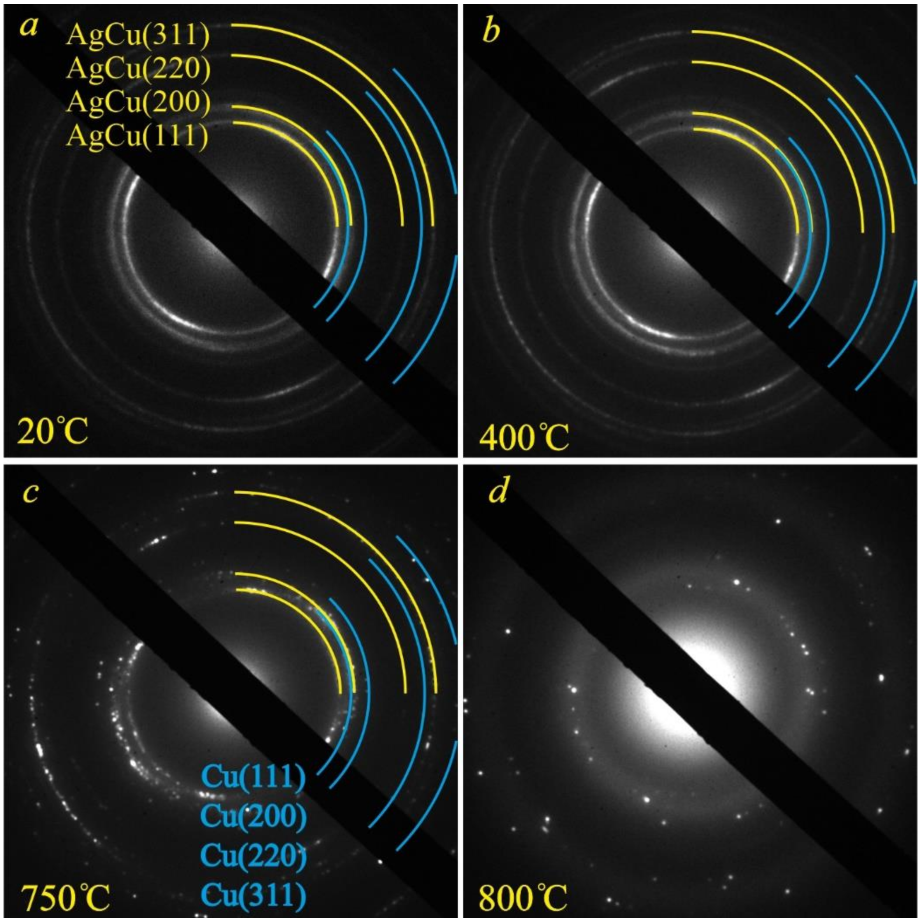

Figure S1. SAED patterns of the Ag – Cu film with a mass thickness of 70 nm at different temperatures.

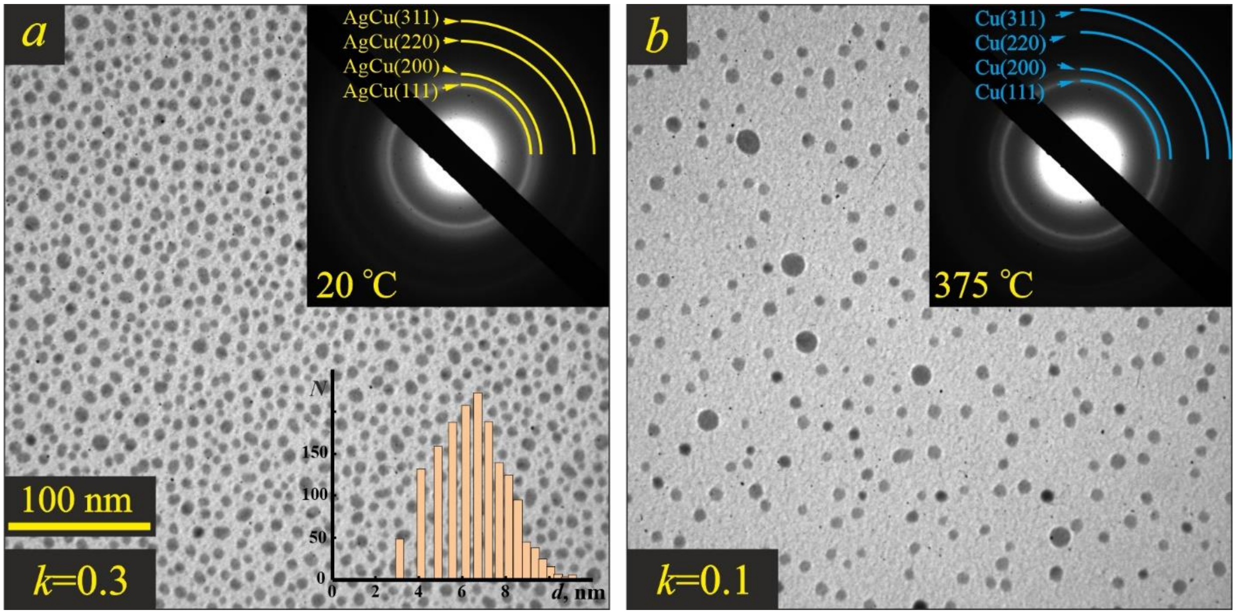

Figure S2. Bright-field TEM images of the Ag–Cu film with a total mass thickness of 1.3 nm at 20 °C (a) and 375 °C (b) along with the corresponding SAED patterns shown in the insets. The size distribution of the islands is shown in the insert in (a). The coverage is specified in the bottom right corner of the image.

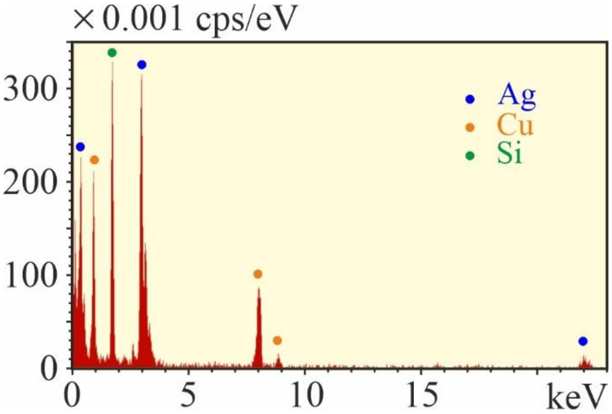

Figure S3. EDX spectrum from the center of the single-phase Ag-Cu nanoparticle shown in Figure 9c.

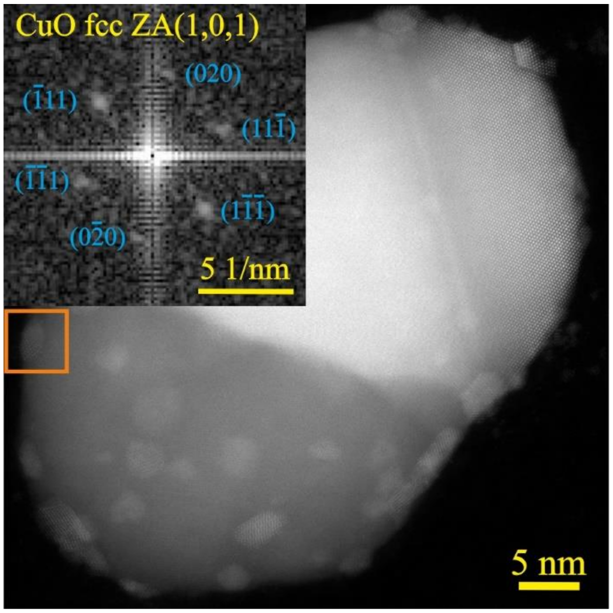

Figure S4. HAADF STEM image of Ag-Cu Janus nanoparticle. Inset shows the fast Fourier transformation from the marked region, revealing fcc structure of CuO.
